# Editorial: Emerging Technologies and Systems for Biologically Plausible Implementations of Neural Functions

**DOI:** 10.3389/fnins.2022.863680

**Published:** 2022-03-31

**Authors:** Erika Covi, Elisa Donati, Stefano Brivio, Hadi Heidari

**Affiliations:** ^1^NaMLab gGmbH, Dresden, Germany; ^2^Institute of Neuroinformatics, University of Zurich, Eidgenössische Technische Hochschule Zürich (ETHZ), Zurich, Switzerland; ^3^CNR—IMM, Unit of Agrate Brianza, Agrate Brianza, Italy; ^4^Microelectronics Lab, James Watt School of Engineering, University of Glasgow, Glasgow, United Kingdom

**Keywords:** memristive devices, neuromorphic circuit, sensors, learning, editorial

The human brain is a complex and fascinating biological machine. With 20 W only, the activity of 100 billion neurons and 3 orders of magnitude more (10^15^) synapses in a volume as small as a shoebox allow us to learn, process, sense, and perceive a vast amount of information from the external environment in real-time. The human brain features a distributed system based on slow and unreliable components. Yet, it is able to learn from experience and compute unstructured data reliably with extreme energy efficiency. Therefore, our brain's real-time and low-power cognitive processes have always been the ultimate ambition in terms of building artificial systems for Edge Information-Extraction and Computing, User-Specific Applications such as Healthcare, Autonomous vehicles, Robotics, and the Internet of Things. Studies on the brain lead to models describing its operating and computational principles, which in turn can be used as a guideline to build new devices, circuits, and systems emulating brain functionality.

Neuromorphic Very Large-Scale Integration (VLSI) circuits model neural networks using a synthetic biology approach whereby they attempt to understand the properties of brain-inspired neural networks by building biologically plausible artifacts that reproduce the physics of the biological systems they model. Neuromorphic circuits can exhibit very slow, biologically plausible, time constants, facilitating the artificial system and/or real-world interaction. Despite the slow time constants, the neuromorphic neural processing chips have fast response times, thanks to a distributed memory, which improves the latency typical of conventional von Neumann architectures. For these reasons, neuromorphic systems can be developed to carry out sensory data analysis and information extraction and solve problems in noisy and uncertain settings and constraint satisfactory problems. In addition, these systems are able to learn from experience, leading to significant progress in the perceptive abilities of e.g., robots, security, and healthcare systems.

Recently, emerging technologies, encompassing memristive and spintronic devices, have been investigated to further improve the memory performance and to complement Complementary Metal Oxide Semiconductor (CMOS) technology, in power-limited neuromorphic systems on edge. Thanks to their excellent performance in terms of high scalability, low latency, low-power operation, and their ability to reversibly change their conductance upon applying proper electrical stimuli, these devices are being researched to emulate artificial synaptic or neural behaviors. Furthermore, their intrinsic physical properties are well suited to implement spike-based time, rate-sensitive operations locally, and support edge-of-chaos dynamics, as well as fundamental computing primitives belonging to biological neurons and synapses.

This Research Topic provides an overview of the avant-garde artificial biologically plausible sensing, computing, and perception paradigms and technologies enabling biologically plausible neuromorphic systems. Contributions cover the following areas:

1. Sensors for biological signals and external environmental stimuli.
2. Emerging devices, circuits, and systems enabling neuromorphic paradigms.
3. Emerging technologies and device models to emulate synaptic plasticity and learning.
4. Biologically plausible models that are implementable in neuromorphic sensing, computing, and perception systems.

The 18 articles in this collection span and merge several disciplines, from the field of engineering to life science and neuroscience, with a significant portion of cross-disciplinary works, as illustrated in Figure 1. Furthermore, this collection provides a good representation of worldwide research, collecting contributions from four continents and 14 countries.

**Figure 1:**
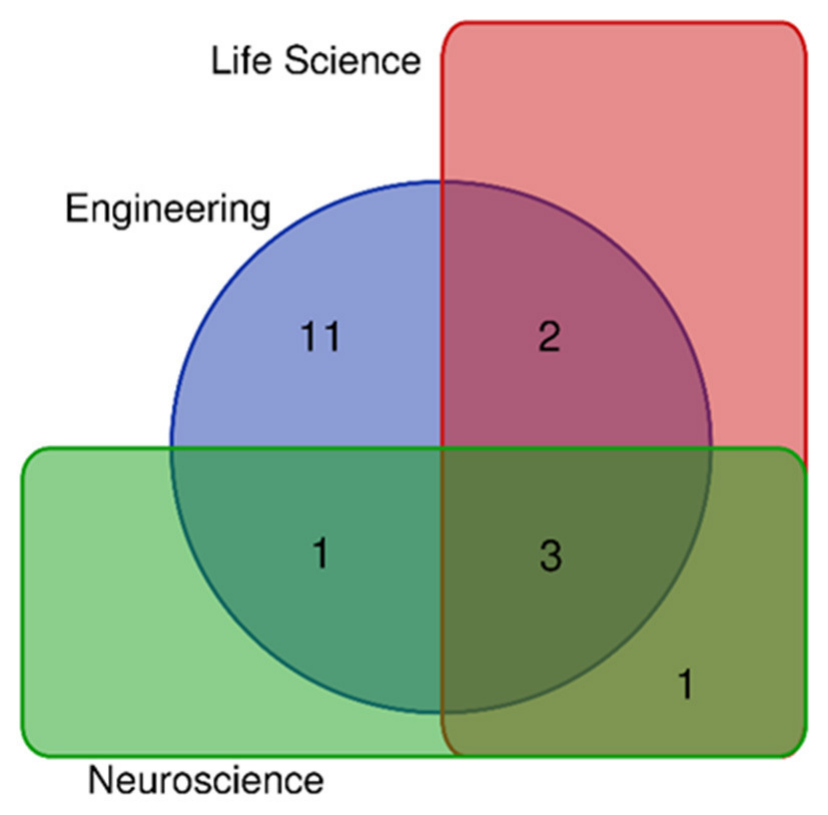
Distribution of the contributions across disciplines.

Among the authors, only 14% are female and the percentage of female first/last authors is 16% each. The data evidence that there is still a long path ahead to achieve gender balance.

## Author Contributions

All authors listed have made a substantial, direct, and intellectual contribution to the work and approved it for publication.

## Conflict of Interest

The authors declare that the research was conducted in the absence of any commercial or financial relationships that could be construed as a potential conflict of interest.

## Publisher's Note

All claims expressed in this article are solely those of the authors and do not necessarily represent those of their affiliated organizations, or those of the publisher, the editors and the reviewers. Any product that may be evaluated in this article, or claim that may be made by its manufacturer, is not guaranteed or endorsed by the publisher.

